# Epidemiology of drowning deaths in the Philippines, 1980 to 2011

**DOI:** 10.5365/WPSAR.2016.7.2.005

**Published:** 2016-11-08

**Authors:** Rammell Eric Martinez, John Juliard Go, Jonathan Guevarra

**Affiliations:** a Office of the WHO Representative in the Philippines, Sta. Cruz, Manila, Philippines.; b Department of Health Promotion and Education, College of Public Health, University of the Philippines Manila.

## Abstract

Drowning kills 372 000 people yearly worldwide and is a serious public health issue in the Philippines. This study aims to determine if the drowning death rates in the Philippine Health Statistics (PHS) reports from 1980 to 2011 were underestimated. A retrospective descriptive study was conducted to describe the trend of deaths caused by drowning in the Philippines from official and unofficial sources in the period 1980 to 2011. Information about deaths related to cataclysmic causes, particularly victims of storms and floods, and maritime accidents in the Philippines during the study period were reviewed and compared with the PHS drowning death data.

An average of 2496 deaths per year caused by drowning were recorded in the PHS reports from 1980 to 2011 (range 671–3656). The average death rate was 3.5/100 000 population (range 1.3–4.7). An average of 4196 drowning deaths were recorded from 1980 to 2011 (range 1220 to 8788) when catacylsmic events and maritime accidents were combined with PHS data. The average death rate was 6/100 000 population (range 2.5–14.2).

Our results showed that on average there were 1700 more drowning deaths per year when deaths caused by cataclysms and maritime accidents were added to the PHS data. This illustrated that drowning deaths were underestimated in the official surveillance data. Passive surveillance and irregular data management are contributing to underestimation of drowning in the Philippines. Additionally, deaths due to flooding, storms and maritime accidents are not counted as drowning deaths, which further contributes to the underestimation. Surveillance of drowning data can be improved using more precise case definitions and a multisectoral approach.

## Introduction

Drowning is the process of experiencing respiratory impairment from submersion/immersion in liquid. It is a serious and neglected public health threat that claims the lives of 372 000 people per year worldwide. (*1*) It is the third leading cause of unintentional injury death, accounting for 7% of all injury-related deaths. More than 90% of these deaths occur in low- and middle-income countries. (*1*) In the Philippines, there were 3044 reported deaths due to drowning in 2010. (*2*) The profile of drowning deaths is expected to vary significantly across the Philippines since the country has diverse hazards, population densities and levels of development.

In the Philippines, there are two national databases that capture accidental drowning: the National Civil Registry and the Online National Electronic Injury Surveillance System (ONEISS). The National Civil Registry captures deaths from accidental drowning and submersion from all health authorities. Both the public and private sectors report to this system as it is required by law. These data are published regularly in the Philippine Health Statistics (PHS) reports. On the other hand, both fatal and non-fatal drownings are captured by the ONEISS, and data in this system are collected only by hospitals (both public and private) that are registered in the system. ONEISS is maintained by the Department of Health. Drowning deaths in the PHS reports include those coded under the category of “accidental drowning and submersion” in the National Civil Registry but not those categorized as cataclysm, including flood, storm and tsunami, intentional drowning deaths or water-transport-related incidents. (*2*) In addition, there are drowning deaths that are not reported or classified due to the remoteness of the incidents. Deaths caused by drowning are likely to be underestimated in the Philippines. This study aims to provide a more comprehensive documentation of drowning deaths in the Philippines from 1980 to 2011.

## Methods

### Study design

A retrospective descriptive study was conducted to describe the number and trend of deaths caused by drowning in the Philippines from official and unofficial sources from 1980 to 2011.

### Data collection

Data about deaths caused by drowning in the Philippines were retrieved from the PHS reports from 1980 to 2011. (*3*) For the deaths related to cataclysmic causes and maritime accidents in the Philippines during this period, a Google search retrieved related literature and reports online. Keywords used for the search include “Philippine typhoons,” “Pacific typhoons,” “Philippine storms,” “capsize ship Philippines,” “maritime accidents” and “maritime disaster in the Philippines.” The search was performed in English. The same search strategy was also applied to retrieve posts specifically on the Wikipedia web site. The first 10 hits in the search results were reviewed by the authors. Related information from these resulting web pages was extracted for analysis. In addition, news from two Philippines local online news agencies (*4*, *5*) was reviewed to retrieve drowning-related information. Information extracted includes the number of deaths related to drowning; cataclysmic events (including flood, storm, typhoon, storm surge and all water-related disasters); and maritime accidents. Only information from 1980 to 2011 was extracted for analysis.

### Data analysis

Data analysis for the deaths caused by drowning was conducted. The estimated number of actual drowning deaths was calculated by summing PHS data with additional deaths from cataclysmic storm, typhoon and maritime accident retrieved from Wikipedia, Google search and news agencies. The estimated death rates were computed based on the projected population retrieved from PHS in the given year.

All analyses were conducted using Excel version 2010 (Microsoft Excel, Redmond, WA, USA).

This paper does not breach issues of confidentiality. All information was validated and considered to be true.

## Results

**Fig. 1:**
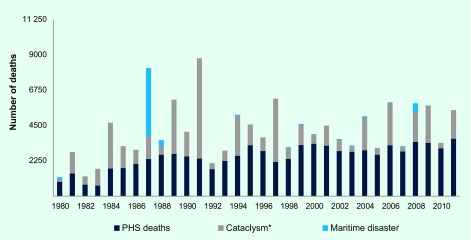
Combined number of drowning and other water-related deaths, Philippines, 1980 – 2011

**Fig. 2:**
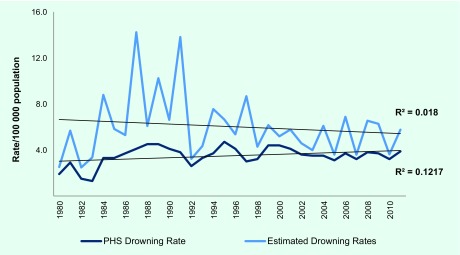
Drowning death rate of the PHS data only and the combined drowning deaths rate*, Philippines, 1980–2011

An average of 2496 deaths caused by drowning per year were recorded in PHS from 1980 to 2011 (range 671–3656) (**Fig. 1**). The average death rate was 3.5/100 000 population (range 1.3–4.7). The highest peak of drowning death rates was in 1995 with the death rate of 4.7 per 100 000 population, followed by 1988 and 1989 (rate = 4.5/100 000 population) and 1999 and 2000 (rate = 4.4/100 000 population) (**Fig. 2**). The death rate plateaued from 2002 to 2011; in 2011, 3656 deaths were caused by drowning (death rate = 3.9/100 000 population).

PHS, Philippine Health Statistics

* include tsunami, cataclysmic storm, flood, exposure to other and unspecified forces of nature (e.g. tidal wave)

PHS, Philippine Health Statistics

* include deaths from tsunami, cataclysmic storm, flood, exposure to other and unspecified forces of nature (e.g. tidal wave)

When PHS data were combined with the number of deaths caused by water-related cataclysmic events and maritime accidents, an average of 4196 deaths per year (range 1220–8788) from 1980 to 2011 was revealed. The average death rate was 6.0/100 000 population (range 2.5–14.2). The highest peaks of death rate for the combined drowning death data were in 1987 and in 1991 (**Fig. 2**). The average number of deaths due to cataclysm was 1515 per year from 1980 to 2011 (range 131–6397), and the average number of deaths due to maritime accidents was 185 per year (range 0–4352) (**Fig. 1**). On average there were 1700 deaths per year from water-related cataclysm and maritime accidents with an average death rate of 2.5/100 000 population. When water-related cataclysmic causes and maritime accidents were added, the average number of annual deaths due to drowning (4196 deaths per year) is 1.68 times the PHS estimate (2496 deaths per year).

## Discussion

Our results showed that on average there were 1700 deaths per year in addition to the PHS data of drowning deaths when cataclysm and maritime accidents deaths data retrieved from other sources were included. This clearly illustrated that drowning deaths were underestimated in the official report. An underestimated report of drowning reflected by the PHS data is likely contributed to neglecting drowning as a serious public health issue. The lack of a comprehensive national drowning prevention strategy also adds to the neglect of this public health issue. The World Health Organization (WHO) Global Report on Drowning (2014) (*1*) recommends that collection of drowning rates and circumstances surrounding drowning are necessary in drowning prevention. Likewise, a strict implementation of death registration is necessary. Use of the WHO verbal autopsy instrument is also useful when underestimation is suspected. (*1*)

One reason for the underestimation is that the definition of drowning deaths in PHS is not comprehensive. The National Civil Registry followed the International Statistical Classification of Diseases and Related Health Problems 10th Revision (ICD-10) to classify drowning cases. (*6*) In ICD-10, the whole range of conditions is classified into mutually exclusive categories. Accidental drowning and submersion were coded as W65-W74, but this category excludes water-transport-related drowning and submersion (coded as V90 and V92) and drowning and submersion caused by cataclysm (coded as X34-X39). Victims of cataclysmic storms (X37), victims of floods (X38) and victims of tsunamis (X34.1) are combined into the category of cataclysm (X34-X39) in PHS but not in the category of drowning and submersion in PHS. Additionally, intentional self-harm by drowning and submersion (X71) is combined into the category of self-harm, and assault by drowning or submersion (X92) is combined into the category of assault. In the future, consolidation of the above-mentioned drowning-related codes into a single category would facilitate estimation of all drowning-related deaths.

Drowning (fatal and non-fatal) is also captured by ONEISS in the Philippines. ONEISS data can be used as the source of information in determining primary cause and risk factors of drowning. (*7*) However, there are limitations for using the ONEISS data as (1) the data are collected by selected hospitals; (2) the system is web-based and hospitals with no or poor access to the Internet will have problems in using the system; (3) drowning events captured by local health clinics are not usually reported; (4) cataclysmic events and water transport accidents are not included; and (5) like other countries in Asia, misclassification of cases could be a problem. (*8*) We did not include ONEISS data in the analysis as basically all the drowning deaths in ONEISS were captured in the National Civil Registry. ONEISS can be improved by considering other sources to collect drowning incidence data. (*9*) Also it is necessary to avoid double-entry of patients referred or transferred from one health facility to another. Additional variables for patient coding can avoid this issue and should be considered. (*10*, *11*)

This study has several limitations. First, the data were limited only to available information collected from the PHS reports and information online. (*3*) Additional drowning death data such as accidental drowning, submersion and other non-specific water-related deaths may have been missed. The results are only a conservative estimate and the actual number of drowning deaths may be even higher. Second, this study only provided yearly data due to the availability of information. With data of higher resolution, trends for drowning deaths could be better presented to determine if seasonality is a contributing factor for drowning deaths. Third, the reliability of data from online media and grey literature was not examined. The deaths captured in media reports may not be the final death tolls as the situations evolved. Some natural disasters and maritime accidents might have been missed.

## Conclusion

When cataclysmic and maritime deaths data from online sources were combined with PHS data, the number of deaths due to drowning per year is 1.68 times the PHS estimate in the Philippines in 1980–2011. This clearly showed that drowning deaths were underestimated in the official surveillance data. Surveillance of drowning data can be improved using more precise case definitions and a multisectoral approach.
